# *Helicobacter pylori sabA* gene is associated with iron deficiency anemia in childhood and adolescence

**DOI:** 10.1371/journal.pone.0184046

**Published:** 2017-08-30

**Authors:** Seiichi Kato, Takako Osaki, Shigeru Kamiya, Xue-Song Zhang, Martin J. Blaser

**Affiliations:** 1 Department of Pediatrics, Tohoku University School of Medicine, Sendai, Japan; 2 Department of Infectious Diseases, Kyorin University School of Medicine, Mitaka, Japan; 3 Departments of Medicine and Microbiology, New York University School of Medicine, New York, NY, United States of America; International Centre for Diarrhoeal Disease Research Bangladesh (icddr,b), BANGLADESH

## Abstract

**Background:**

Gastric *Helicobacter pylori* colonization leads to iron deficiency anemia (IDA), especially in children and adolescents. However the pathogenesis is poorly understood.

**Objective:**

We sought to identify specific *H*. *pylori* genes involved in IDA development, by comparing bacterial genome-wide expression profiling in patients affected or not.

**Methods:**

*H*. *pylori* were isolated from four children with IDA and four from matched controls without IDA. Based on these isolates, cDNA microarrays under iron-replete or depleted conditions were systematically performed to compare gene expression profiles at the whole genome level. Real-time reverse-transcription (RT-) PCR and protein assays were performed for further assessing the profile differentiation of the identified *H*. *pylori* IDA-associated genes.

**Results:**

We identified 29 and 11 genes with significantly higher or lower expression in the IDA isolates compared to non-IDA isolates, respectively. Especially notable were higher expression of *sabA* gene encoding sialic acid-binding adhesin in the IDA isolates, which was confirmed by real-time RT-PCR study. Moreover, iron-depletion *in vitro* led to up-regulation of *fecA1* and *frpB1* genes and down-regulation of *pfr*, as predicted. Known iron-regulated genes such as *fur*, *pfr*, *fecA*, and *feoB* did not significantly differ between both groups. The IDA isolates had significantly higher expression of vacuolating cytotoxin gene *vacA* than non-IDA isolates, consistent with the results of VacA protein assays. There were no significant differences in bacterial growth value between IDA and non-IDA isolates.

**Conclusions:**

It is likely that *H*. *pylori* carrying high expression of *sabA* causes IDA, especially in children and adolescents who have increased daily iron demand. In addition, it is possible that several host-interactive genes, including *vacA*, may play a synergistic role for *sabA* in IDA development.

## Introduction

*Helicobacter pylori* is closely associated with the development of peptic ulcer disease [1]. Long-term *H*. *pylori* colonization also leads to atrophy and intestinal metaplasia, pre-disposing to adenocarcinoma in adults [2]. Many *H*. *pylori* characteristics have been related to disease risk, including the *cag* island encoding both Type IV secretion system and *cagA*, particular *vacA* genotypes and genes encoding outer membrane proteins (OMPs), including blood group antigen-binding adhesin BabA, sialic acid-binding adhesin SabA, and outer membrane inflammatory protein OipA [3–5].

*H*. *pylori* also induces iron deficiency and iron deficiency anemia (IDA), especially in children and adolescents [6]. Three principal hypotheses have been advanced to explain these observations. First, IDA may be caused by blood loss from *H*. *pylori*-induced gastroduodenal lesions. However, most *H*. *pylori*-infected children do not have hemorrhagic mucosal lesions [1]. A second hypothesis is based on *H*. *pylori*-induced gastric atrophy, with resulting impaired gastric acidity in adults [7]. Non-heme iron accounts for >80% of dietary iron in developed countries and reduction from the ferric (Fe^+++^) to ferrous (Fe^++^) form is essential for intestinal absorption; both gastric acid and ascorbic acid play roles in this process [8]. In *H*. *pylori-*infected children, however, significant gastric atrophy is rare [9] and gastric acid secretion is not impaired [10].

Third, competition between *H*. *pylori* and humans for iron availability could lead to IDA [6]. Many bacteria secrete high-affinity ferric chelators (siderophores), and take up ferric iron-siderophore complexes via specific OMPs, mediated by the energy-transducing TonB-ExbB-ExbD system [11]. Although *H*. *pylori* does not have an identified siderophore or its specific receptor, the microorganism expresses proteins associated with iron metabolism, including the ferric uptake regulator (Fur), high-affinity transporters of ferrous iron (FeoB) and ferric dicitrate (FecA), and non-heme iron-containing ferritin (Pfr) [12]. Although *pfr* or *feoB* variation has not been implicated [13, 14], comparative proteomic analysis suggests that particular *H*. *pylori* polymorphisms could promote IDA [15]. Several iron-responsive OMPs may play roles in *H*. *pylori* heme uptake [16, 17].

Knowledge of iron-uptake mechanism in *H*. *pylori* is limited. Using a whole-genome DNA microarray, we sought to identify specific *H*. *pylori* IDA-associated genes, by comparing bacterial gene expression profiling in patients with or without IDA.

## Materials and methods

### *H*. *pylori* strains

*H*. *pylori* strains were isolated from four children aged 13–16 years (two male/two female) with IDA (strains TH1, TH2, TH4, and TH6) and from four age- and sex-matched controls without IDA (control strains TH3, TH5, TH7, and TH8). All four IDA patients had hypochromic microcytic anemia (range of serum hemoglobin values, 6.1–7.4 g/dL) with low serum iron (range, 6–12 μg/dL) and ferritin values (range, 1.1–4.9 ng/mL). In control subjects, ranges of serum hemoglobin, iron, and ferritin values were 13.9–15.1 g/dL, 80–132 μg/dL and 17.5–34.4 ng/mL, respectively. Fecal occult blood tests using anti-hemoglobin antibodies were negative in all eight subjects. Routine biochemical examinations, including liver function tests and total serum protein and albumin, were normal. The patients with and without IDA were referred to Tohoku University Hospital because of moderate or severe IDA or with gastrointestinal symptoms, such as epigastric pain or persistent nausea. All subjects underwent upper gastrointestinal endoscopy and gastric biopsies were obtained. For the IDA patients, upper gastrointestinal endoscopy was performed to examine for gastrointestinal bleeding. Endoscopy showed chronic gastritis without mucosal bleeding in all eight patients; the biopsy specimens were histologically studied and used for diagnosis of *H*. *pylori* infection including culture. After the presence of *H*. *pylori* was confirmed, the four patients without IDA were regarded as *H*. *pylori*-positive non-IDA controls. The IDA patients received a 7-day course of eradication therapy with lansoprazole, amoxicillin and clarithromycin [18]. The patients also received iron supplementation for two months. In all 4 IDA patients, success of *H*. *pylori* eradication was confirmed by ^13^C-urea breath test [19] performed 4 weeks after the completion of eradication therapy. In each case, IDA was substantially improved and no recurrence was observed in any patients at 6 to 12-month follow-up. *H*. *pylori* culture from the gastric biopsy specimens was performed under microaerobic conditions (5% O_2_, 15% CO_2_, and 80% N_2_) at 37°C for 72 hour on Mueller-Hinton agar (Eiken, Tokyo, Japan) supplemented with 5% defibrinated sheep blood (Sigma Chemical Co., St Louis MO).

### Ethics statement

This study was reviewed and approved by the Ethics Committee of Tohoku University School of Medicine. Written informed consent was obtained from parents of the child participants on their behalf.

### Gastric histology

Biopsy specimens obtained from the gastric body and antrum were examined in both IDA and control patients according to the updated Sydney system [20]. The degrees of *H*. *pylori* density, neutrophil (activity) and mononuclear cell infiltration (inflammation), atrophy, and intestinal metaplasia were analyzed.

### cDNA microarray

For liquid cultures, *H*. *pylori* cells were inoculated to OD_595_ of 0.05–0.10 and cultured in Brucella broth containing 7% horse serum with shaking under microaerobic conditions. Total RNA was purified from each culture using ChargeSwitch® Total RNA Cell Kits (Invitrogen Corporation, Carlsbad CA). The integrity of the purified RNA was verified using 1% agarose gel electrophoresis. The *Helicobacter pylori* 4-plex gene expression microarray (Roche NimbleGen Madison WI) was used, which represents 1,576 protein-coding genes in the *H*. *pylori* 26695 genome (accession number NC_000915, 72,000 features/array). A total of 10 μg of bacterial RNA was processed and labeled as per the standard NimbleChip protocol. Briefly, RNA was converted into cDNA using the SuperScript II cDNA Conversion Kit (Invitrogen). Double-stranded cDNA was random-prime labeled with Cy3-nonamers and hybridized to the microarray for 16 hour at 42°C. The arrays were scanned at 5 μm resolution using a GenePix 4000B microarray scanner (Molecular Devices, Sunnyvale CA). Data were extracted from scanned images using NimbleScan^TM^ software. Quantile normalization was performed across replicate arrays, and RMA (Robust Multiple Average) analysis was performed to generate gene expression values, which were compared between IDA and control strains.

### Study of expression of iron-regulated genes

To examine gene regulation by ferric iron, cDNA microarray analysis also was performed for liquid cultures of *H*. *pylori* under iron-restricted conditions. Two IDA strains (TH4 and TH6) and two control strains (TH3 and TH7) were cultured in Brucella broth containing 5% horse serum with and without 50 μM of the iron chelating reagent deferoxamine mesylate (DFM) under microaerobic conditions as above. Total RNA was purified from exponential-phase (OD_600_ = 0.5–0.9) broth cultures and cDNA microarray was performed as indicated above. For each isolate, gene expression values were compared for RNA samples from liquid culture with and without 50 μM DFM.

### Assessing gene expression by real-time RT-PCR

Expression of *sabA*, *sabB*, *coaX*, and *vacA* in the eight *H*. *pylori* strains was determined by real-time RT-PCR using the SYBR Green method (**Table 1**). Absolute quantitation analysis was performed with the ABI real time system 7500 using a standard curve for each gene. The primer sets which were previously reported were used for *vacA* [21].

**Table 1:** Primers used for PCR in this study.

Gene	Primer	Sequence	Reference
*sabB*	HP0722-F	CCCAACTGGCTTCGTTAAAA	This study
	HP0722-R	TGGGTATCATCGCCTTAATGT	This study
*sabA*	HP0725-F	CCAACAACATTGAGCTGGTC	This study
	HP0725-R	TTGCAARATRGGTATCATCG	This study
*coaX*	HP0682-F	AAGTGGGGGCGATGTATGCT	This study
	HP0682-R	CACGCGCAATAAATGCTCTTTG	This study
	HP0682-F2	CAAGGGGTGAATTAGGCAAA	This study
	HP0682-R2	CCAAGCATGCCCAAAACTAT	This study
*vacA*	VA1-F	ATGGAAATACAACAAACACAC	Ref. ^21^
	VA1-R	CTGCTTGAATGCGCCAAAC	Ref. ^21^
	VA3-F	GGTCAAAATGCGGTCATGG	Ref. ^21^
	VA3-R	CCATTGGTACCTGTAGAAAC	Ref. ^21^
	VA4-F	GGAGCCCCAGGAAACATTG	Ref. ^21^
	VA4-R	CATAACTAGCGCCTTGCAC	Ref. ^21^

### Assessing *vacA* genotype, expression, and VacA synthesis

#### *vacA* genotyping

To evaluate the *vacA* genotype of each *H*. *pylori* isolate, the *vacA* signal region (s1 or s2 alleles) with the products differentiated on the basis of molecular size (259bp or 286bp) and the middle regions (m1 or m2 alleles) were amplified (**Table 1**), as described [21]. Genomic DNA was extracted from each strain using the MagExtractor kit (Toyobo, Osaka).

#### Immunoblot analysis

A 72-hour culture of each isolate was centrifuged at 8,500 g and passaged through a 0.45 μm pore size membrane filter (Millipore) to remove bacteria, and supernatants analyzed for the VacA protein. In brief, 15 μL of sample was mixed with an equal volume of loading buffer (EZ apply, ATTO) and boiled for 3 min. The sample was electrophoresed through a 12.5% sodium dodecyl sulfate (SDS)-polyacrylamide gel. Migrated proteins then were electrophoretically transferred (0.14 mA for 1 hour) onto a PVDF membrane. After incubation in blocking buffer (5% skim milk in PBS), the PVDF membrane was treated with anti-VacA primary antibody (b-300, sc-25790, Santa Cruz Biotechnology Inc.) at room temperature for 1 hour with gentle shaking. Then membranes were washed with PBS containing 0.1% Tween 20 (T-PBS) and then incubated with horseradish-peroxidase-conjugated goat anti-rabbit IgG (Santa Cruz Biotechnology Inc.) for 1 hour at room temperature. After incubation, the membrane was washed 5 times with T-PBS, and specific bands were detected using the Amersham ECL Plus Western blotting system (GE Healthcare), according to the manufacturer’s protocol.

#### Cytotoxic assay

Assay for VacA activity was essentially as described [22]. In brief, the 72-hour-culture filtrate obtained from each *H*. *pylori* isolate was diluted with RPMI-1640 (Sigma) containing 10% heat-inactivated fetal bovine serum (RPMI-FBS) in serial two-fold dilutions. AGS cells (5 x 10^4^) were seeded into 96-well plates in RPMI-FBS and incubated at 37°C in 5% CO_2_. After 24-hour incubation, cell culture medium was removed from each well, and replaced with 200 μL of each sample. After a further 24-hour incubation, the extent of cytoplasmic vacuolation in AGS cells was observed microscopically. The titer of the vacuolating toxin was determined as the highest dilution of the culture filtrate showing vacuolation of >10% of cells and the reciprocal number of the dilution (toxin titer) was used for statistical analysis.

### Statistical analyses

In cDNA microarray, a ≥3.5-fold difference in gene expression values was defined as significant. Student’s *t–*test with Bonferroni correction were also performed and *p* <0.05 was considered statistically significant. In the real-time RT-PCR study, the mean of those relative values was used for statistical analysis by the Wilcoxon rank sum test.

## Results

### Histological study

In the gastric body and antrum, there were no differences in the histologic features between the IDA and control groups (**S1 Table**). Intestinal metaplasia was not observed in any biopsy site in either group. Thus, as expected, differences in gastric histology did not explain the cause of the IDA.

### Bacterial growth of IDA and control isolates

**Fig 1:**
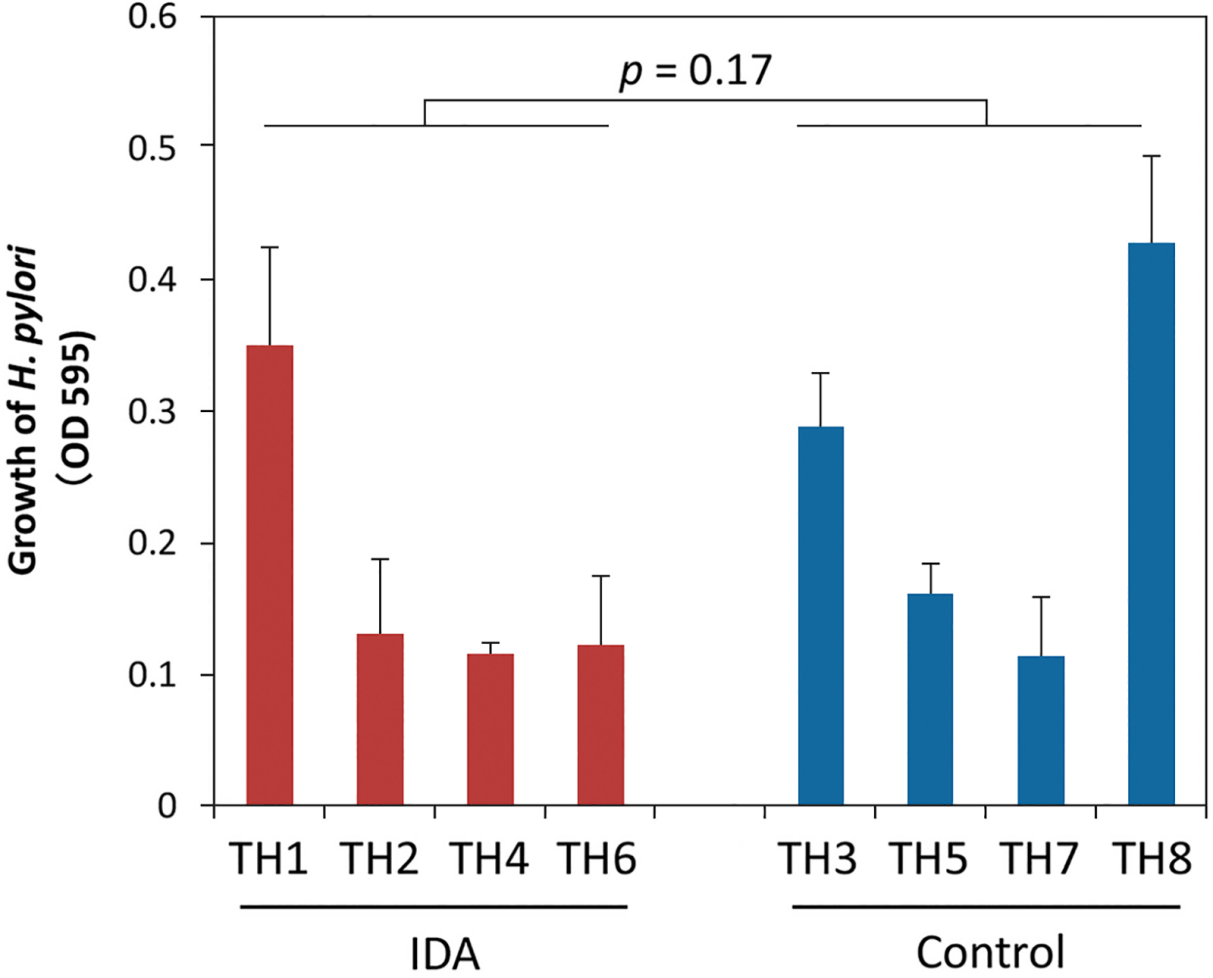
Growth of *H*. *pylori* strains isolated from IDA or control patients. Each strain was inoculated into Brucella broth containing 5% horse serum at an initial OD_595_ 0.05. The growth of *H*. *pylori* was measured (OD_595_) by microplate photometer after 24-hour incubation. The Mean±SD 24-hour growth value was measured based on three independent determinations for each strain. Bacterial growth was not significantly different between the IDA and control strains (*p* = 0.17; Wilcoxon rank sum test).

Each strain was inoculated into Brucella broth containing 5% horse serum at an initial OD_595_ = 0.05. There were no significant differences in bacterial growth value (three independent determinations) measured by microplate photometer (OD_595_) after 24-hour incubation between IDA (Mean ± SD, 0.18±0.11) and control groups (0.25±0.13) (*p* = 0.17) (**Fig 1**).

### Reliability of microarray data

**Fig 2:**
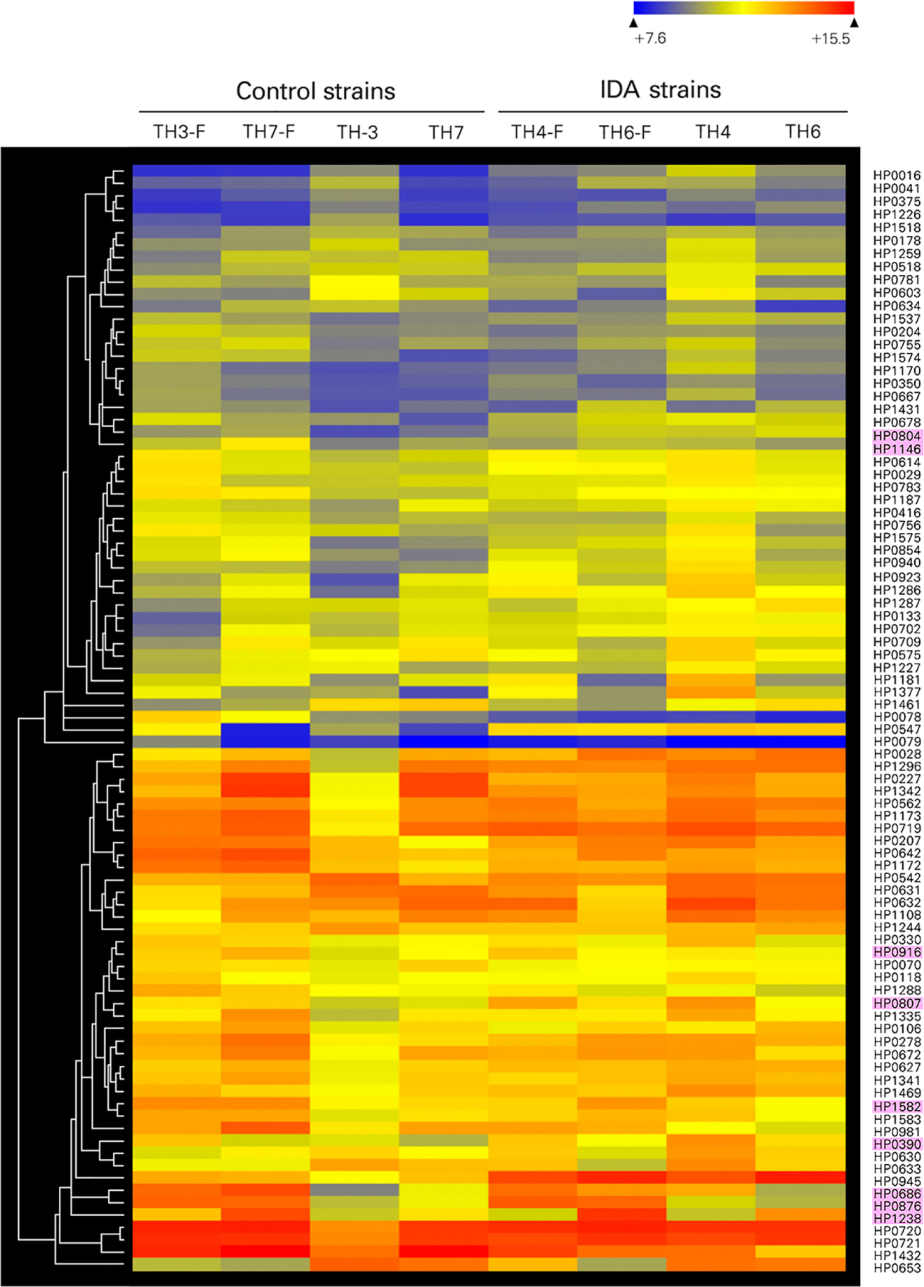
Hierarchical cluster analysis of gene expression alteration of control *H*. *pylori* strains under iron-replete and limited conditions. Each strain was cultured with DFM (iron-limited condition, indicated as TH3-F, TH7-F, TH4-F, and TH6-F) or without DFM (iron-replete condition, indicated as TH3, TH7, TH4, and TH6). The genes with significant differences in expression were included in hierarchical clustering analysis using average linkage and Euclidean dissimilarity methods. Genes with a Fur-binding site consensus sequence are indicated in pink.

Comparing the gene expression profiles of *H*. *pylori* isolates with microarray data clearly indicated differential expression of several genes known to be Fur-regulated under iron-limited conditions (**Fig 2** and **S2 Table**). Under iron-restricted conditions, *pfr* was down-regulated while *fecA1* (HP0686) encoding iron (III) dicitrate transport protein and *frpB1* genes (HP0876) encoding iron-regulated OMP were up-regulated in both the IDA and control strains (**Table 2**), consistent with prior observations of iron-regulated expression [23]. Expression of *fur* and *feoB* did not significantly differ between iron-replete and -restricted conditions in either group. The expression of *amiF* was up-regulated 4.3-fold by iron starvation, confirming a prior report [24]. In total, results of iron-regulated gene expression under iron-restricted conditions confirm the microarray data in the present study.

**Table 2:** Expression of *H*. *pylori* genes known to be related to iron metabolism.

		Iron replete		Iron restricted/replete	
		condition:		ratio in:	
Gene designation and category	Gene name	IDA/control ratio ^a^	*p* value	Control	IDA
Non-heme iron ferritin:					
HP0653	*pfr*	1	1	0.1 ^b^	0.3 ^b^
Ferric uptake regulator:					
HP1027	*fur*	0.9	1	1	1.9
Iron (Fe^++^) transport protein:					
HP0687	*feoB*	1.3	1	1.3	0.8
Iron (Fe^+++^) transport protein:					
HP0807	*fecA1*	0.9	1	11.2^b^	4.0 ^b^
HP0686	*fecA2*	0.9	1	1.8	1.2
HP1400	*fecA3*	0.8	0.52	0.6	0.8
Iron-regulated OMP:					
HP0876	*frpB1*	0.2	0.004	8.4 ^b^	17.3 ^b^
HP0915	*frpB2*	0.6	0.61	1	1.3
HP0916	*frpB3*	0.9	0.61	1	0.9
HP1512	*frpB4*	1.1	1	1	1

^a^ Values shown are mean of triplicate determinations.

^b^ *p* values in the control versus IDA group are <0.001.

### Possible IDA-related genes in microarray analysis

**Fig 3:**
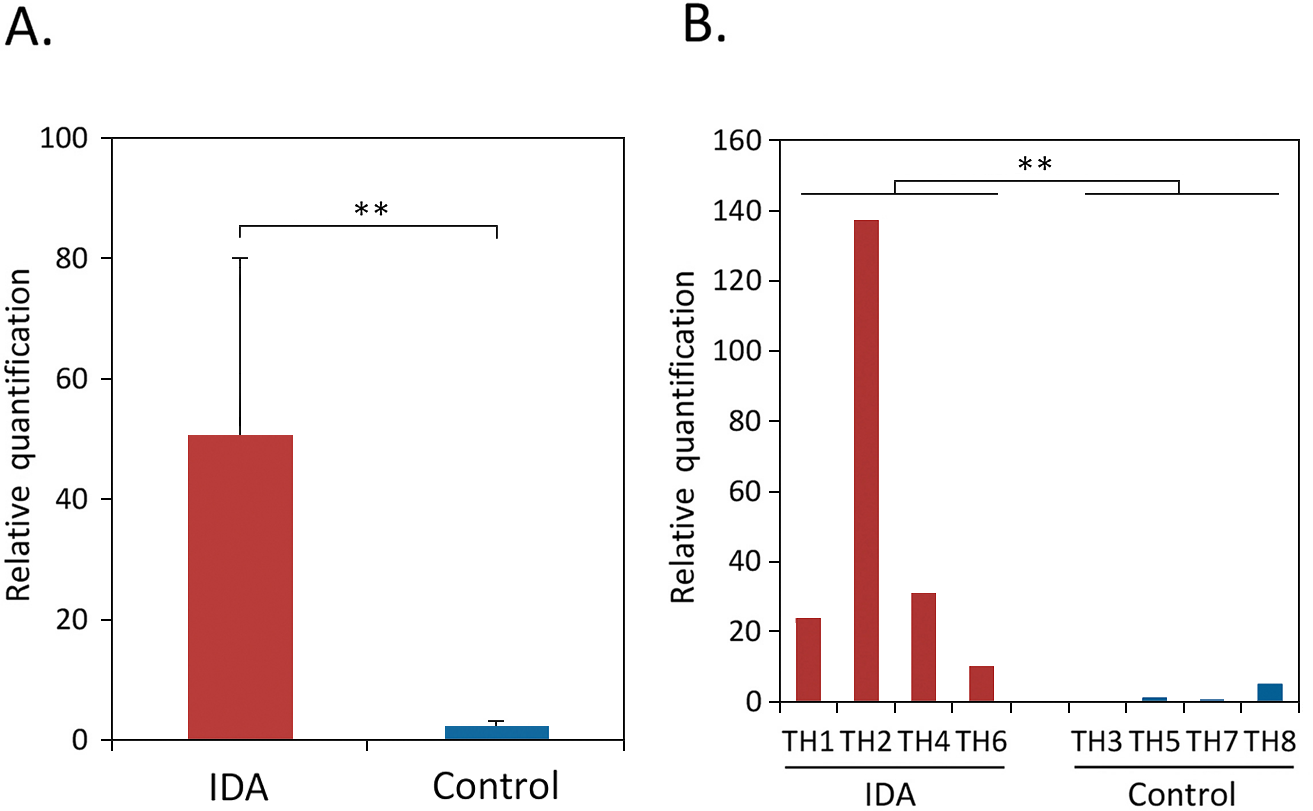
Transcription of *sabA* gene in IDA or control strains by real-time RT-qPCR. Panel A: overall expression (relative quantitation) of *sabA* in the four IDA and four control strains, respectively. Panel B: expression (relative quantitation) of *sabA* in the eight strains studied each. Expression of *sabA* was normalized in relation to house-keeping gene *gyrA*. For relative quantitation, *H*. *pylori* TH5 (control) strain was used as control, because expression value of the strain was the least (defined as 1.0). Panel A: results are shown as the Mean ± SE for triplicate determinations. ** *p* = 0.029, using Wilcoxon rank sum test.

By microarray analysis, 29 genes had significantly higher expression in the IDA group than in the controls (**Table 3**). These included the genes encoding two OMPs, SabA and its homolog SabB. The real-time RT-qPCR analysis indicated that *sabA* expression was detected in all four IDA and three control strains; relative expression in the IDA group was significantly higher than in the controls (*p* = 0.029) (**Fig 3**). In contrast, expression of *sabB* was detected by RT-qPCR only in one control (TH8) and two IDA strains (TH2 and TH6). Although *coaX* showed 84-fold higher expression in the IDA group (**Table 3**), the expression in the real-time RT-qPCR analysis was not demonstrated in one IDA (TH4) and two control strains (TH3 and TH7); there was no significant difference between both groups (data not shown).

**Table 3:** *H*. *pylori* genes with higher expression^a^ in IDA strains.

Gene designation	Gene name			Expression ratio	
and category		Predicted function		(IDA / control)	*p* value
Cell envelope:					
HP0722	*sabB* (HopO)	adhesin		10.9	< 0.001
HP0725	*sabA* (HopP)	adhesin		3.7	< 0.001
HP0217	*cgtA*	LPS synthesis		3.6	0.002
DNA metabolism:					
HP0142	*mutY*	DNA repair		5.4	< 0.001
HP0462	*hsdS*	restriction system		7.3	< 0.001
HP0463	*hsdM*	restriction system		10.6	< 0.001
HP0592	*res*	restriction system		3.6	0.003
Cellular processes:					
HP0682	*coaX*	CoA synthesis		84.0	0.003
HP1510	*folB*	folic acid synthesis		3.7	< 0.001
HP1511		predicted *frpB-*like protein		3.6	< 0.001
Other categories:					
HP0428	*terY*	phage/colicin		3.5	0.02
HP0414		transposase A		4.3	0.018
HP0731	*leoA*	toxin production		3.9	< 0.001
HP1000		partition protein A		6.0	< 0.001
Hypothetical proteins:					
HP0016		unknown		5.2	< 0.001
HP0059		unknown		3.9	0.001
HP0120		unknown		6.0	< 0.001
HP0199		unknown		4.1	0.001
HP0435	COG1112 superfamily DNA and RNA-helicase	unknown		3.7	0.022
HP0436	COG3843, *virD2*	unknown		5.9	0.015
HP0489		unknown		3.6	0.03
HP0583		unknown		4.5	< 0.001
HP0999		unknown		4.6	0.021
HP1116		unknown		3.5	0.047
HP1165	*tetA*	unknown		4.3	< 0.001
HP1334	COG1432	unknown		3.8	< 0.001
HP1371	type III restriction enzyme R protein	unknown		4.1	0.001
HP1388		unknown		4.2	0.036
HP1589	COG4735	unknown		3.8	0.004

^a^ As determined by microarray analysis (see Methods).

On the other hand, 11 genes showed significantly lower expression in the IDA group, including *ceuE1*, encoding an iron ABC transporter, and *frpB1*, *e*ncoding a heme and hemoglobin-binding membrane protein (**Table 4**). Five of the 29 genes with significantly higher expression in the IDA strains are related to DNA-processing (**Table 3**), as are two of the 11 genes with lower IDA-strain expression (**Table 4**).

**Table 4:** *H*. *pylori* genes with lower expression in IDA strains.

Gene designation	Gene name				Expression ratio			
and category		Predicted function			(IDA / control)		*p* value	
Iron transport:								
HP0876	*frpB1*	Iron-regulated outer membrane protein			0.22		0.022	
HP1561	*ceuE1*	Iron(III) ABC transporter			0.02		< 0.001	
Cell envelope:								
HP1125	*palA* (omp18)	Peptidoglycan associated lipoprotein precursor			0.03		< 0.001	
DNA metabolism:								
HP1209	*iceA*	Ulcer-associated gene, restriction endonuclease			0.15		0.003	
HP1352		Type II methylase			0.20		< 0.001	
Hypothetical proteins:								
HP0168		Unknown			0.17		< 0.001	
HP 0187		Unknown			0.16		< 0.001	
HP0556		Unknown			0.21		< 0.001	
HP0641		Unknown			0.06		< 0.001	
HP1289		Unknown			0.09		0.008	
HP1324		Unknown			0.07		< 0.001	

### Expression of *H*. *pylori* genes related to host interaction

**Fig 4:**
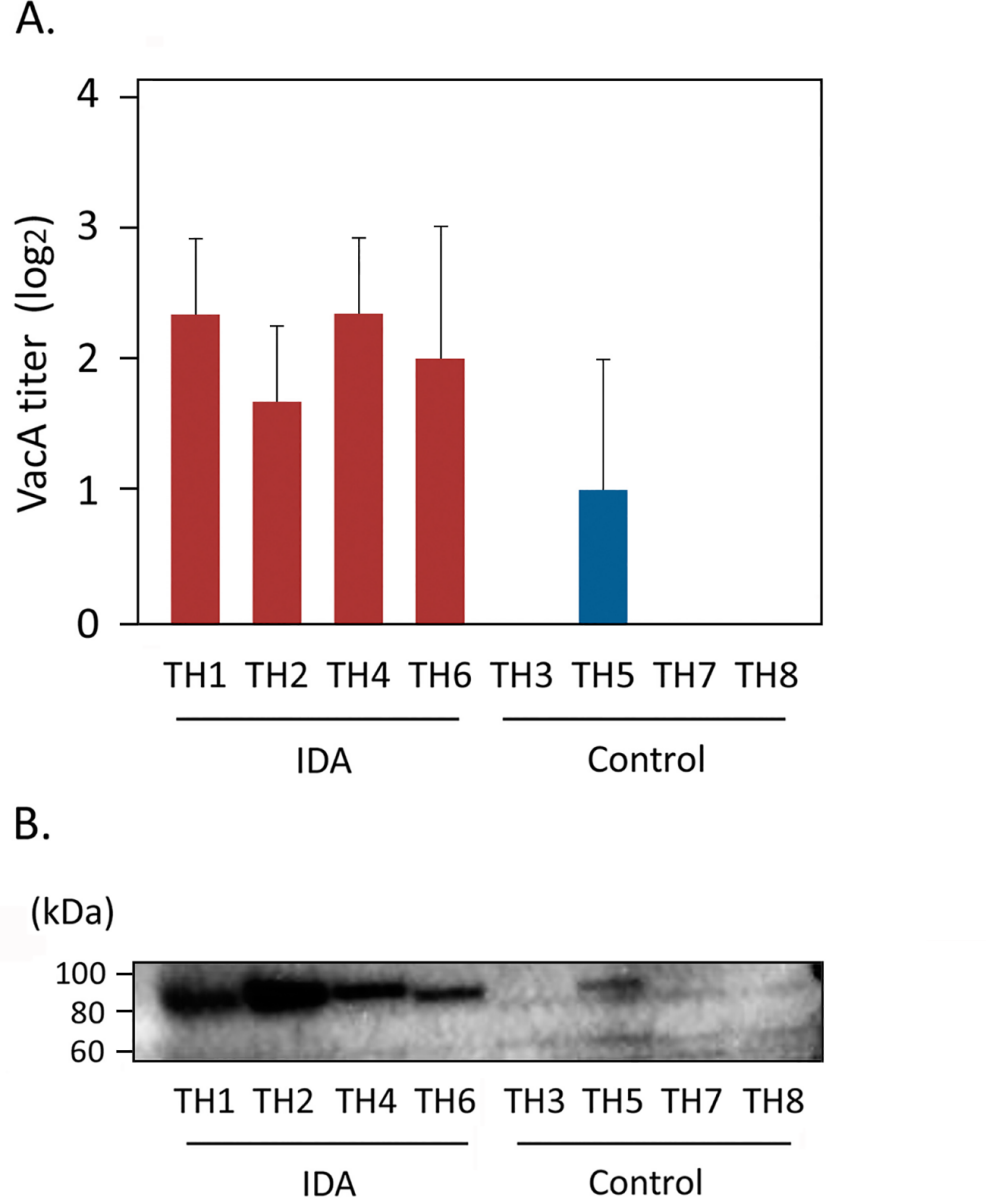
VacA activity in IDA and control strains. Panel A: *H*. *pylori* cytotoxic activity. Filtrate obtained from 72-hour cultures of each *H*. *pylori* strain was serially diluted and added to AGS cell cultures. After 24-hour incubation, the final dilution with visible vacuoles was determined microscopically and reciprocal number of the dilution used for calculation. Panel B: Immunoblot analysis of culture filtrate of the eight *H*. *pylori* strains.

In the microarray analysis, *vacA* in the IDA strains was highly expressed (3.2-fold difference) compared to the controls. There were no differences in *vacA* expression of the IDA group between iron-replete and -restricted conditions. By RT-qPCR, there were no significant differences in *vacA* expression between IDA and control strains (*p* = 0.77). However, in both cytotoxic assays and immunoblot analysis, all four IDA and one control isolate secreted detectable amounts of the VacA protein (**Fig 4**). In the *vacA* genotype analysis, three IDA and one control strains carried the s1/m1 alleles. One IDA and two control strains carried the s1 allele but the mid region allele was not identified. One control strain had s1/m2 alleles. Real-time RT-qPCR showed that mRNA of *vacA* (signal region) was detected in all four IDA strains but only one control strain carrying the s1/m1 alleles in the *vacA* genotype analysis. In the microarray analysis, all eight isolates studied carried the *cag* pathogenicity island (data not shown).

The expression of *cagN* and *babB* were significantly lower in the IDA group than in the control (**Table 5**). For the IDA strains, there was no difference in the expression of these genes between iron-replete and restricted culture conditions, nor were there differences for *sabA* and *sabB*. In total, these results indicated that IDA strains differ from the control strains in expression of several genes related to host interaction, including *vacA*.

**Table 5:** Expression of *H*. *pylori* OMPs and genes associated with host interaction.

		Iron replete		Iron restricted/replete	
Gene designation	Gene	Ratio in:		Ratio in:	
and category	name	IDA/Control	*p* value ^a^	Control	IDA
Major OMP family:					
HP1243	*babA*	1.1	1	1	1.1
HP0896	*babB*	0.4	<0.001	0.7	0.8
HP0638	*oipA*	1.3	1	0.8	1.1
HP0725	*sabA*	3.7	<0.001	0.9	1
HP0722	*sabB*	10.9	<0.001	0.9	1.1
Host interaction:					
HP0243	*napA*	1.4	0.85	1.2	0.7
HP0887	*vacA*	3.2	<0.001	0.7	0.7
HP0547	*cagA*	0.2	0.18	1.6	0.9
HP0538	*cagN*	0.5	<0.001	0.9	0.9

^a^ Student’s *t–*test with Bonferroni correction.

## Discussion

By isolating *H*. *pylori* strains from children with IDA and from age- and sex-matched control without IDA, and then systemically comparing gene expression profiles under iron-replete or depleted conditions, we identified a group of *H*. *pylori* genes with significantly different expression between the IDA and control groups. IDA was associated with *sabA*, encoding the sialic acid-binding adhesin (SabA), which mediates *H*. *pylori* binding to human gastric epithelial cells during colonization [25]. Binding to glycosylated epithelial cells is essential for *H*. *pylori* to persistently colonize and adherence to gastric mucosa is dependent on SabA [25]. SabA also has been associated with gastroduodenal disease [26], playing a role in non-opsonic activation of human neutrophils [27], and contributing to bacterial persistence [28]. *H*. *pylori sabA* expression is phase-variable via both a homopolymeric T tract in its promoter region [29], and multiple CT tracts in the coding region [26]. Its product, SabA, can interact with multiple sialylated host receptors on neutrophils, sialyl-Lewis^x^ [28], sialyl-Lewis^a^ [25], laminin, and zinc-alpha 2-glycoprotein [25, 27, 28]. Our identification of higher expression in IDA suggests a link between *H*. *pylori*-signaling to host cells and iron deficiency.

The IDA strains had significantly higher *vacA* expression than in controls, consistent with the results of VacA protein assays (**Fig 4**). *H*. *pylori* isolates carrying *vacA* s1/m1genotypes show higher vacuolating activity than those carrying other genotypes, and are more frequently associated with gastric pathologies [3]. One hypothesis is that strains with particular *vacA* genotypes may more effectively obtain iron under iron-depleted conditions through their interactions with host cells. The VacA protein also interferes with the T cell/interleukin-2 signaling pathway and efficiently blocks T cell proliferation [30]. A prior study using mRNA expression profiling suggested that VacA escapes host immune defenses by differentially regulating the expression of host genes related to immune evasion [31]. It is possible that host-interactive genes including *vacA* and *sabA*, may play synergistic roles in *H*. *pylori-*associated IDA development by enhancing chronic inflammation. The VacA protein induces cytotoxicity that can damage host cells [21, 32]. This may lead to increased iron concentrations in tissue. We hypothesize that *H*. *pylori* cells that highly express *sabA* take up iron via SabA, an outer membrane protein; however, examining this hypothesis will require further study.

It is possible that reduced iron in tissue results in overexpression of *sabA*. The IDA and control patients, who are age- and sex-matched, have histological chronic gastritis to a comparable extent. There are no other obvious differences between the groups except for the presence or absence of IDA. The microarray study showed that in both IDA and control strains, several known iron-regulated genes were expressed, as reported [23, 24]. Moreover, our study showed that IDA strains consistently expressed *sabA* to a high degree in both replete and restricted iron conditions (**Table 5**). Significantly higher expression of *sabA* was also demonstrated by real-time RT-PCR. These data are consistent with the hypothesis that high-level expression of SabA is involved in the causation of IDA, rather than the consequence. However, this hypothesis must be tested directly in future studies.

One limitation of the present study is a small sample size, which may not be sufficient to fully examine the findings. In the present study, we compared the strains from *H*. *pylori*-positive children with and without IDA, and we restricted the analysis to moderate or severe IDA but not mild IDA. We believe that mild IDA is not suitable for narrowing down the candidate genes, because mild IDA can be induced by causes other than *H*. *pylori*. It is not common for children to present for medical attention with moderate or severe *H*. *pylori*-related IDA, in whom both intestinal bleeding and insufficient dairy iron intake have been excluded. Therefore, we studied strains from only four of these highly targeted patients. Even with such a small group, we did demonstrate that there is a significant difference in *sabA* expression between the strains from the IDA and control groups.

The endoscopic and histological diagnosis in all of the patients with and without IDA is chronic gastritis induced by *H*. *pylori*, and the only consistent difference between cases and controls was the presence or absence of IDA. Therefore, we believe that the strains from non-IDA patients are suitable as controls for IDA strains. In addition, we used a 3.5-fold difference in gene expression as the threshold for considering significant differences between the groups of strains. The choice was empirical because at the threshold of 3.5-fold difference, there were 40 implicated genes, but at 3.0, there were 71 genes. To minimize false discovery, we used the higher threshold for increased stringency.

*H*. *pylori frpB1* and *ceuE1*, both Fur-regulated [33], are involved in iron uptake. That *H*. *pylori* isolates from IDA patients express *frpB1* and *ceuE1* at lower levels than control strains may reflect that *H*. *pylori* has ample iron in the IDA milieu. Another gene with lower expression in the IDA strains, *palA* (HP1125), encodes a peptidoglycan-associated lipoprotein that enhances Th1 responses by activating dendritic cell maturation [34]. Whether the low *palA* expression contributes to IDA or is a consequence is uncertain.

Few studies have focused upon iron-associated *H*. *pylori* genes in the context of IDA pathogenesis. Protein sequences encoded by *pfr* do not differ in isolates from patients with or without IDA [13], nor has *feoB* been implicated [14]. In addition to these genes, the present microarray data do not show involvement of the major iron-regulated genes, including *fur* and *fecA*, in the pathogenesis of *H*. *pylori*-induced IDA, nor is there an association between *nikR*, encoding a nickel-dependent regulator protein, and IDA (data not shown).

In iron-replete environments, *H*. *pylori* expresses a single *fecA3* (HP1400) and *frpB4* (HP1512) OMP [12]. Under iron-restriction, however, all putative iron transport proteins are expressed [35], consistent with our finding of greater transcription of *fecA1* (HP0807) and *frpB1* (HP0876) under iron-restriction than repletion. That IDA strains express less *frpB1* (HP0876) than control strains (**Table 4**), may reflect more iron availability in IDA patients.

Under iron-replete culture conditions, *H*. *pylori* isolates from IDA patients have been reported to show more rapid growth and enhanced uptake of both ferrous and ferric ions compared to those from non-IDA patients [36]. However, the similar growth of IDA and control strains under iron-replete conditions that we observed suggests that other hypotheses should be considered.

In childhood and adolescence, it is well known that daily iron demand increases especially for catch-up growth. In addition, more enhanced iron supply is necessary for athletes or pubescent females. Choe *et al*. reported that adolescent female athletes may have *H*. *pylori*-related IDA, suggesting that increased daily iron demand is important in childhood [37].

## Supporting information

S1 Table Gastric histology in IDA and control patients. (DOCX)

S2 Table Gene designations in *H*. *pylori* genes present in hierarchical cluster analysis. (DOCX)
