# Supplementary material for: Helicobacter pylori sabA gene is associated with iron deficiency anemia in childhood and adolescence
Source: PLoS One. 2017 Aug 30;12(8):e0184046. doi: 10.1371/journal.pone.0184046 (PMC5576686; doi:10.1371/journal.pone.0184046)
Supplement: S1 Table — (DOCX) [file pone.0184046.s001.docx]

| **S1** **Table. Gastric histology in IDA and control patients** | | | | | | |
| --- | --- | --- | --- | --- | --- | --- |
|  |  | Histological parameter (antrum/corpus)ª | | | | |
| Strain no. | Age  /sex | Inflammation | Activity | *H.pylori*  density | Atrophy | Intestinal  metaplasia |
| IDA patients: | | | | | | |
| 1 | 13/M | 3/2 | 1/1 | 2/1 | 1/0 | 0/0 |
| 2 | 15/F | 3/1 | 2/0 | 2/1 | 0/0 | 0/0 |
| 4 | 16/M | 3/2 | 2/2 | 3/2 | 1/0 | 0/0 |
| 6 | 14/F | 1/1 | 1/1 | 1/1 | 0/0 | 0/0 |
| Median |  | 3/1.5 | 1.5/1 | 2/1 | 0.5/0 | 0/0 |
| Control patients: | | | | | | |
| 3 | 16/M | 3/2 | 1/0 | 2/1 | 1/0 | 0/0 |
| 5 | 14/F | 3/1 | 2/1 | 2/1 | 0/0 | 0/0 |
| 7 | 13/M | 2/1 | 1/1 | 1/1 | 0/0 | 0/0 |
| 8 | 15/F | 2/1 | 1/0 | 1/1 | 0/0 | 0/0 |
| Median |  | 2.5/1 | 1/0.5 | 1.5/1 | 0/0 | 0/0 |
| ª According to the updated Sydney system. | | | | | | |
