# Supplementary material for: Helicobacter pylori sabA gene is associated with iron deficiency anemia in childhood and adolescence
Source: PLoS One. 2017 Aug 30;12(8):e0184046. doi: 10.1371/journal.pone.0184046 (PMC5576686; doi:10.1371/journal.pone.0184046)
Supplement: S2 Table — (DOCX) [file pone.0184046.s002.docx]

| **S2 Table. Gene designations in *H. pylori* genes present in Hierarchical cluster** | |
| --- | --- |
| **analysis**^a^ | |
| Gene number | Description |
| HP0029 | dethiobiotin synthetase (*bioD*) |
| HP0070 | urease accessory protein UreE |
| HP0079 | omp3 (*horA*) |
| HP0106 | cystathionine gamma-synthase |
| HP0133 | serine transporter (*sdaC*) |
| HP0178 | spore coat polysaccharide biosynthesis proteinE |
| HP0207 | ATP-binding protein (*mpr*) |
| HP0227 | omp5 |
| HP0278 | guanosine pentaphosphate phosphohydrolase (*gppA*) |
| HP0330 | ketol-acid reductoisomerase |
| HP0390 | adhesin-thiol peroxidase (*tagD*) |
| HP0416 | cyclopropane fatty acid synthase (*cfa*) |
| HP0518 | conserved hypothetical secreted protein |
| HP0542 | cag pathogenicity island protein *cag21* |
| HP0547 | cag pathogenicity island protein *cag26* |
| HP0562 | *rpsU* (30S ribosomal protein S21) |
| HP0630 | modulator of drug activity (*mda66*) |
| HP0631 | quinone-reactive Ni/Fe hydrogenase, smallsubunit (*hydA*) |
| HP0632 | quinone-reactive Ni/Fe hydrogenase, largesubunit (*hydB*) |
| HP0633 | quinone-reactive Ni/Fe hydrogenase, cytochrome bsubunit (*hydC*) |
| HP0634 | quinone-reactive Ni/Fe hydrogenase (*hydD*) |
| HP0642 | NAD(P)H-flavin oxidoreductase |
| HP0653 | nonheme iron-containing ferritin (*pfr*) |
| HP0672 | aspartate aminotransferase |
| HP0686 | iron(III) dicitrate transport protein (f*ecA*) |
| HP0755 | molybdopterin biosynthesis protein (*moeB*) |
| HP0804 | bifunctional 3,4-dihydroxy-2-butanone4-phosphate synthase |
| HP0807 | iron(III) dicitrate transport protein (*fecA*) |
| HP0854 | guanosine 5'-monophosphate oxidoreductase |
| HP0876 | iron-regulated outer membrane protein (*frpB*) |
| HP0916 | iron-regulated outer membrane protein (*frpB*) |
| HP0923 | omp22 (*hopK*) |
| HP0940 | amino acid ABC transporter, periplasmic bindingprotein (*yckK*) |
| HP0981 | exonuclease VII-like protein (*xseA*) |
| HP1036 | 7, 8-dihydro-6-hydroxymethylpterin-pyrophosphokinase |
| HP1108 | pyruvate flavodoxin oxidoreductase subunit gamma |
| HP1170 | glutamine ABC transporter, permease protein(*glnP*) |
| HP1172 | glutamine ABC transporter |
| HP1181 | multidrug-efflux transporter |
| HP1226 | coproporphyrinogen III oxidase |
| HP1227 | cytochrome c553 |
| HP1238 | *amiF*(formaidase) |
| HP1244 | *rpsR* (30S ribosomal protein S18) |
| HP1287 | transcriptional regulator (*tenA*) |
| HP1296 | *rpsM* (30S ribosomal protein S13) |
| HP1335 | tRNA(5-methylaminomethyl-2-thiouridylate)-methyltransferase |
| HP1341 | siderophore-mediated iron transport protein (*tonB*) |
| HP1342 | omp29 |
| HP1377 | flagellar assembly protein FliW |
| HP1431 | dimethyladenosine transferase |
| HP1432 | histidine and glutamine-rich protein |
| HP1461 | cytochrome c551 |
| HP1574 | riboflavin synthase subunit alpha |
| HP1575 | ABC transporter |
| HP1582 | pyridoxine 5'-phosphate synthase |
| HP1583 | *pdxA* |
| ^a^ http://genodb.pasteur.fr/ and http://www.ncbi.nlm.nih.gov/gene. | |
